# Commentary on “The Association Between Serum Uric Acid Levels and the 10‐Year Prospective Risk of Dyslipidemia: A Cohort Study From Healthy Heart Project (YHHP)”

**DOI:** 10.1002/hsr2.71196

**Published:** 2025-08-18

**Authors:** Ebrahim Hejazian, Mohammad Barary, Soheil Ebrahimpour

**Affiliations:** ^1^ Department of Neurosurgery, School of Medicine Babol University of Medical Sciences Babol Iran; ^2^ Student Research Committee, Department of Pharmacology, School of Medicine Shahid Beheshti University of Medical Sciences Tehran Iran; ^3^ Infectious Diseases and Tropical Medicine Research Center, Health Research Institute Babol University of Medical Sciences Tehran Iran

Dear Editor,

Mohammadi et al. report that higher baseline serum uric acid (SUA) predicts 10‐year incident dyslipidemia in 693 adults from the Yazd Healthy Heart Project (YHHP) [1]. While the cohort adds much‐needed longitudinal data from the Middle East, several design and analytic issues limit the strength of the causal claim:

1. **High attrition and selection bias:**
  More than one‐third of eligible participants were lost before analysis (7% deaths, 27% missing data). If loss to follow‐up was related to baseline SUA or lipid status, hazard ratios could be over‐ or underestimated. Transparent comparison of baseline characteristics between retained and excluded participants and use of multiple imputation would reduce this threat [2].
2. **Single‐time‐point exposure with possible reverse causation:**
  SUA was measured only at enrollment. Renal function, diet, and alcohol intake, which are determinants of both SUA and lipids, may have changed during the decade‐long follow‐up. Repeated SUA measurements or time‐updated covariates are required to confirm temporal precedence and mitigate regression‐dilution bias [3].
3. **Residual and unmeasured confounding:**
  Models adjusted for body‐mass index, blood pressure, and fasting glucose, yet omitted renal function, insulin resistance indices, dietary patterns, urate‐lowering therapy, diuretics, and corticosteroids, all known to influence SUA and lipids [4]. The inclusion of these variables, or at the very least, sensitivity analyses using propensity weighting, would clarify whether SUA is an independent risk marker or merely a surrogate for broader metabolic dysfunction.
4. **Sex‐specific disparity needs formal interaction testing:**
  The association was restricted to men, but the authors did not report a sex × SUA interaction term. Demonstrating heterogeneity is essential before proposing male‐specific screening thresholds.
5. **Outcome misclassification:**
  Dyslipidemia was defined by a single lipid panel or treatment status after 10 years. Transient elevations and changes in therapy intensity were not captured. Serial lipid measurements would provide a more robust endpoint and allow assessment of dose‐response [5].
6. **Generalizability:**
  YHHP participants are urban Iranians. Dietary purine intake, alcohol use, and access to lipid‐lowering drugs differ markedly from those of other populations. Extrapolating the quartile thresholds (≥ 5.37 mg/dL in men) to non‐Iranian settings, therefore, risks ecological fallacy.

In summary, we suggest that future Iranian cohorts should collect repeated SUA and lipid measures, adjust for renal function, medications, and diet, apply multiple imputation for missing data, and test formal interactions by sex and menopausal status. Such improvements will determine whether lowering SUA can truly prevent dyslipidemia or whether it simply tags individuals with an adverse metabolic milieu.

## Author Contributions

**Ebrahim Hejazian:** conceptualization, investigation, writing – original draft. **Mohammad Barary:** investigation, writing – original draft, writing – review and editing. **Soheil Ebrahimpour:** writing – original draft, investigation, supervision.

## Conflicts of Interest

The authors declare no conflicts of interest.

## Transparency Statement

All authors have read and approved the final version of the manuscript. Soheil Ebrahimpour had full access to all of the data in this study and takes complete responsibility for the integrity of the data and the accuracy of the data analysis.

## Data Availability

Data sharing is not applicable to this article as no new data were created or analyzed in this study.
